# Tracing Monotreme Venom Evolution in the Genomics Era

**DOI:** 10.3390/toxins6041260

**Published:** 2014-04-02

**Authors:** Camilla M. Whittington, Katherine Belov

**Affiliations:** 1 School of Biological Sciences, The University of Sydney, Camperdown, NSW 2006, Australia; 2 Faculty of Veterinary Science, The University of Sydney, Camperdown, NSW 2006, Australia; E-Mail: katherine.belov@sydney.edu.au

**Keywords:** crural system, echidna, genome, platypus, toxin, transcriptomics

## Abstract

The monotremes (platypuses and echidnas) represent one of only four extant venomous mammalian lineages. Until recently, monotreme venom was poorly understood. However, the availability of the platypus genome and increasingly sophisticated genomic tools has allowed us to characterize platypus toxins, and provides a means of reconstructing the evolutionary history of monotreme venom. Here we review the physiology of platypus and echidna crural (venom) systems as well as pharmacological and genomic studies of monotreme toxins. Further, we synthesize current ideas about the evolution of the venom system, which in the platypus is likely to have been retained from a venomous ancestor, whilst being lost in the echidnas. We also outline several research directions and outstanding questions that would be productive to address in future research. An improved characterization of mammalian venoms will not only yield new toxins with potential therapeutic uses, but will also aid in our understanding of the way that this unusual trait evolves.

## 1. Introduction: Mammalian Venom

Venom has evolved independently across multiple vertebrate and invertebrate lineages [1]. The reptiles are perhaps the best known group of venomous vertebrates, and the venom of many species is well characterized. In contrast, the venomous mammalian lineages have been largely unstudied until recently. Based on the recent expanded definition of venom as a physiologically or biochemically disruptive substance that is secreted from a specialized gland and delivered via a specialized delivery system [1], there are four lineages of venomous mammals. These include the Insectivora (the short-tailed shrew *Blarina brevicauda*, the European water shrew *Neomys fodiens*, the Mediterranean water shrew *Neomys anomalus*, and the Hispaniolan solenodon *Solenodon paradoxus*, with some indication that other members of this order may also be venomous (reviewed in [2])), the Chiroptera (the common vampire bat *Desmodus rotundus*, the hairy-legged vampire bat *Diphylla ecaudata*, and the white-winged vampire bat *Diaemus youngi* [3]), the Primates (slow lorises *Nycticebus* sp. (reviewed in [4])), and the Monotremata (platypus *Ornithorhynchus anatinus*).

Mammalian venom has been poorly studied for two reasons. First, mammalian toxin characterization (and subsequent antivenin development) has received less attention compared to reptile or invertebrate toxins because of the rarity of life-threatening human envenomation. Second, mammalian venom tends to be available only in small quantities, making identification of the toxic components difficult [5]. Recently, the increasing availability of genomic techniques has resulted in renewed possibilities for the characterization of mammalian venom. This review aims to highlight the current state of knowledge and propose hypotheses of venom evolution in one mammal lineage, the monotremes.

## 2. Venom in Ancient Monotremes

There are five living species of monotreme currently recognized: the platypus *Ornithorhynchus anatinus*, the short-beaked echidna *Tachyglossus aculeatus*, and the long-beaked echidnas *Zaglossus attenboroughi*, *Z. bartoni*, and *Z. bruijnii* [6]. The monotremes have a plethora of unusual features compared to other mammals, including a reptilian-like skeleton, maintenance of a low internal body temperature, lactation without nipples, and, famously, egg-laying rather than live birth.

The current estimate for when monotremes diverged from other mammals is 166 million years ago (MYA) [7]. Although the echidnas (family Tachyglossidae) are thought to have diverged from the platypus lineage (family Ornithorhynchidae) (reviewed in [8,9,10]) ~32 MYA [10], ancient monotremes were much more diverse than extant members monotremes [8], and monotreme evolutionary rates were potentially slow [9]. It is thus difficult to extrapolate the features of the basal monotreme and it should not be assumed that it was platypus-like (reviewed in [8]).

In extant monotremes, venom delivery systems are located in the hind limbs. Unfortunately, as much of the monotreme fossil record is in the form of tooth and jaw fragments (reviewed in [9]), the basal form of the monotreme venom system is unknown. The known monotreme fossil record begins in the Early Cretaceous (~110–115 MYA, reviewed in [8]), with the first fossils of modern monotremes appearing in the Pleistocene (1.78 MYA onwards, reviewed in [8]). Given the presence of either functional or regressed venom systems in all extant monotremes, as will be discussed in Section 3 and Section 4, and recent molecular data indicating that at least one platypus venom component arose ~192 MYA, prior to the divergence of platypuses and echidnas [11], the most recent common ancestor of platypuses and echidnas was probably also venomous. The precise ancestral role of mammalian venom systems, and thus the factors shaping the evolution of these systems, remains unclear. In contrast to many other venomous species, modern monotremes do not utilize their venom systems in relation to prey capture or digestion (reviewed in [12]). The functional or vestigial delivery systems present in extant monotremes of both sexes indicate a probable defensive function for the basal venom system. Venom may have been used to ward off potential predators, representing a distinct survival advantage for the ancient monotremes.

**Figure 1:**
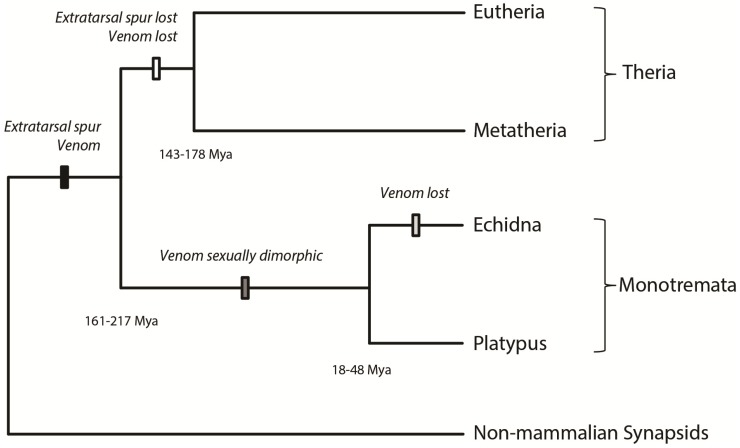
A phylogenetic representation of extratarsal spur and associated venom evolution in mammalian taxa. Divergence date estimates of Phillips *et al.* [10] are used.

Although no fossil evidence of these structures has been found, ancestral monotremes likely had venom delivery systems very similar to that of extant monotremes (see Section 3.1), in the form of an extratarsal spur consisting of *cornu calcaris* (the spur, which is covered by a keratinous sheath) and *os calcaris* (the supporting bone). Interestingly, there are several fossils of non-monotreme mammals with evidence of a potential venom delivery system similar to that of the monotremes. These include fossils with what have been interpreted as extratarsal spurs [3,13], and fossils with an *os calcaris* that may have supported an extratarsal spur [14]. It is thus possible that the extant monotreme extratarsal spur is plesiomorphic, having been retained in monotremes but lost in the therians [14,15]. This implies that many early mammals may have had spurs, and possibly associated venom glands, as a defensive mechanism (Figure 1), although additional fossil evidence is required to resolve this.

## 3. Derived Venom System: The Platypus

### 3.1. The Crural System

**Figure 2:**
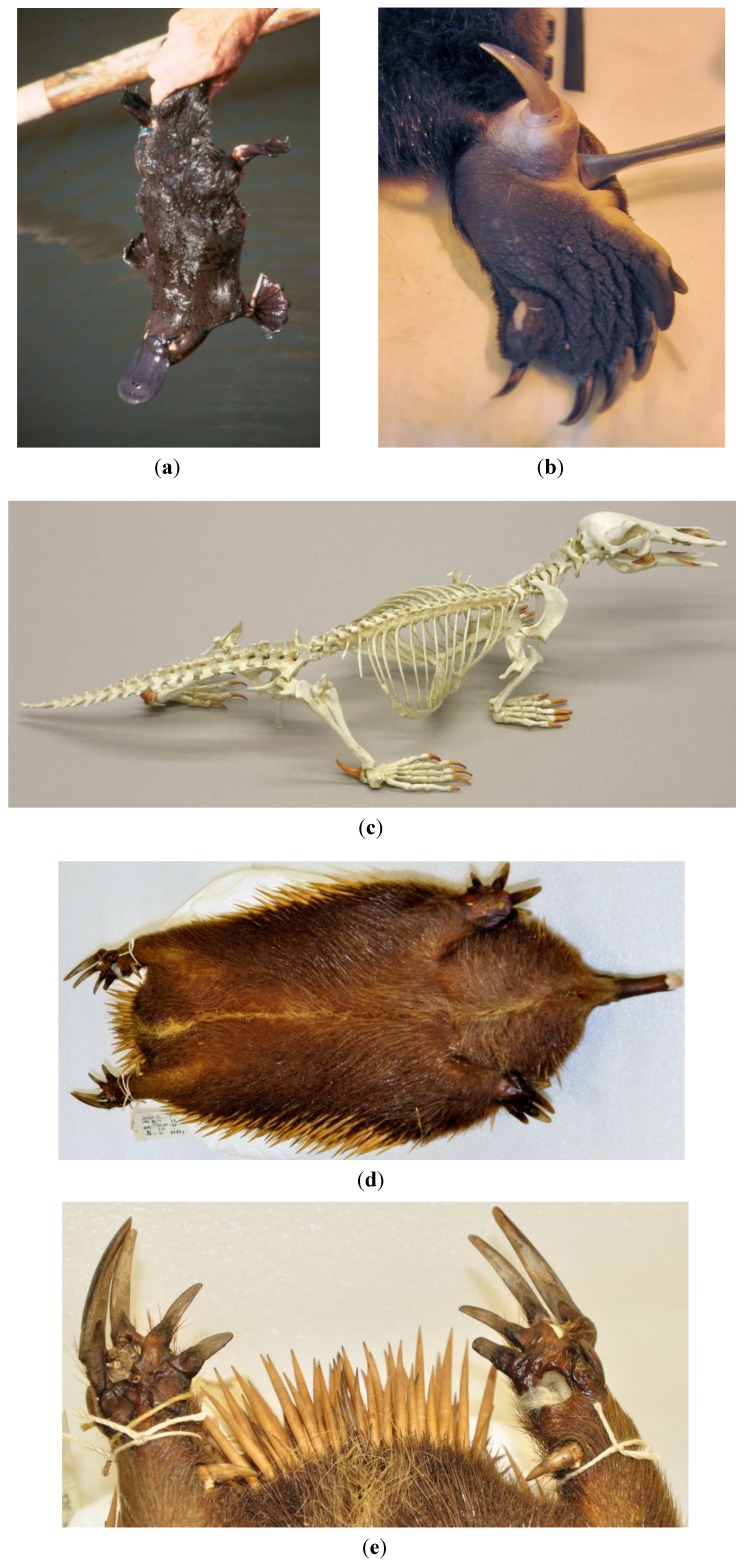
(**a**) Platypus (*Ornithorhynchus anatinus*) (Richard J. Whittington^©^); (**b**) Spur of an adult male platypus, ~15 mm long (found postmortem; forceps are used to erect spur); (**c**) Resin cast of a male platypus skeleton, displaying prominent spurs on the hind limbs (Bone Clones^©^); (**d**) Ventral surface of a preserved male *Tachyglossus* echidna specimen, showing spurs pointing inwards on each hind leg; (**e**) Detail of the spurs in the *Tachyglossus* specimen shown in (**d**).

The sole extant representative of the derived form of the monotreme venom system is the semi-aquatic, semi-fossorial platypus (*O. anatinus*) (Figure 2). The venom apparatus is known as the crural system, because it is in the hind limb, and consists of paired crural venom glands connected by venom ducts to an extratarsal spur on each hind leg [16]. The crural glands are derived from modified apocrine sweat glands and migrate during development from the inner surface of each thigh to their final position on the dorsocaudal surface of the pelvis [17,18].

Uniquely, the venom system in the platypus is sexually dimorphic and appears only in males. A small and non-functional spur sheath develops in females, but is lost by the time they reach adulthood [16]. Venom production occurs at maturity in males, with venom production and gland size vastly increasing during the breeding season [17]. In contrast, during the non-reproductive season, the venom gland secretory epithelium is inactive and the gland regresses [17]. Platypus behavior also changes, with males displaying little aggressive use of their spurs outside the breeding season [17].

The male platypus spur is a hollow keratinous sheath with a *cornu calcaris* core; the sheath develops within a chalky outer conical structure which gradually wears away to reveal a sharp point [16]. The spur is supported by the *os calcaris* (this bone is absent in females [18]), and is attached to tendon and muscle to allow erection of the spurs prior to envenomation [14,17]. Platypus envenomation is rarely observed; the animals wrap their hind legs around and drive the sharp spurs into the victim. The animals are able to hang by their spurs, requiring manual disengagement [19]. Although platypuses have up to 4 mL of venom available to be injected at any one time [17], in practice, the small diameter of the spur aperture (~0.2 mm) and associated high pressure required for injection mean that injected venom volumes around 100 µL are predicted [5].

### 3.2. Venom Function

Early naturalists speculated that male platypuses used their spurs to grip the female during mating [20], but there is little to no evidence to support this hypothesis [16]. A prey capture or digestive function can also be ruled out, as the platypus diet consists mainly of benthic invertebrates [16]. A defensive function is possible, but in this case presumably the spurs would be retained in both sexes. Although foxes and dogs kill platypuses, the species has very few native predators (occasional exceptions are crocodiles, Tasmanian devils and raptors) [16,21]. Perhaps, as discussed in Section 2, this was an ancestral function of the venom, which has since been recruited for other uses once the selective pressure of predation was lifted.

The venom system in extant platypuses undoubtedly serves some purpose, as the venom produces strong pharmacological effects (see Section 3.3). As venom is generally metabolically expensive to produce (reviewed in [22]), its retention implies some utility. The restriction of venom to adult male breeding season platypuses suggests that the venom has a reproductive function, such as use in male-male competition and territory defense during the reproductive period. Healed spur marks in wild platypuses suggest that intraspecific envenomation does occur and is not fatal [16]. Envenomated male platypuses are observed to experience temporary limb paralysis [17], which might be sufficient to either deter territory encroachment and/or to temporarily prevent mating by competitors. Platypus venom may thus be an important component of male reproductive fitness.

### 3.3. Platypus Venom Composition: Pharmacological and Proteomic Characterization

There are few formal clinical reports of platypus envenomation of humans. Envenomation is known to produce the following physiological effects: swelling; a long lasting and excruciating pain that cannot be relieved with conventional painkillers, including morphine [19]; nausea, cold sweats, and lymph node swelling [17]; high erythrocyte sedimentation rates and low total serum protein and albumin levels [19]; and muscle wasting [19]. There have been no recorded human fatalities. Anecdotal evidence suggests that envenomation can kill dogs [20]. There is no antivenin available.

There is a dearth of research into platypus venom compared to that of other animals, despite the potential for drug discovery using newly identified venom compounds [23]. Until recently, platypus venom research was limited to a few biochemical and pharmacological studies, and the venom was poorly characterized due to the difficulty in obtaining samples for study [5]. Early pharmacology studies injecting platypus venom into rabbits produced intravascular coagulation, a drop in blood pressure (likely through vasodilation), hemorrhagic edema, and death [24,25]. Further research identified mild proteolytic activity, histamine release, and cutaneous anaphylaxis in laboratory animals [17]. *In vitro* experiments revealed that platypus venom produces smooth muscle relaxation [26], feeble hemolysis [25], proteolysis [26], and cation currents in cells [27] or cation channels in artificial lipid bilayers [28]. *In vivo*, these effects may disrupt ion concentrations and cause edema, nerve firing, and pain.

Subsequent high performance liquid chromatography (HPLC) studies of platypus venom revealed several peptides and proteins of unknown function [26,29,30]. Until the advent of genomic technologies only three types of peptides had been identified and fully sequenced: the particularly biologically active C-type natriuretic peptides (OvCNPs) [31], the abundant defensin-like peptides (OvDLPs, [30]), and nerve growth factor (OvNGF, Torres A and Kuchel PW, unpublished data; described in [32]). A venom peptide isomerase and a hyaluronidase were also found, but not fully sequenced [26]. The peptide isomerase catalyzes the conversion from l- to d-form of the second amino acid from the N-terminus of both an OvDLP and an OvCNP [33]; the d-form of the amino acid may confer prolonged stability and therefore toxin efficacy in the victim [34]. These components of platypus venom have similarity to known toxins in other venomous animals, and have unknown function [5,12].

### 3.4. Platypus Venom Composition: Genomic Insights

The sequencing of the platypus genome in 2008 [7] provided an unprecedented resource for platypus venom research, and has resulted in a vast increase in our knowledge of platypus toxins. Identification of toxin gene sequences allowed investigations into their tissue expression patterns to facilitate an understanding of their functions and evolution [35,36]. The most notable advance came with the next-generation sequencing of a platypus venom gland transcriptome. This representation of the genes expressed in the tissue reveals a number of predicted new platypus toxins [37]. The putative toxins are categorized into thirteen families: Serine protease, Stonustoxin-like, Kunitz type protease inhibitor, Zinc metalloproteinase, Latrotoxin-like, CRiSP (cysteine rich secretory protein), Sea anemone cytolytic toxin-like, Unknown (IG domains), Mamba intestinal toxin-like, C-type lectin domain-containing, Sarafotoxin-like, VEGF (vascular endothelial growth factor), and DNAse II. These are similar to toxins across a wide range of other venomous animals, including fish, reptiles, spiders, and marine invertebrates. Although the molecules found in platypus venom have not been functionally tested, it is possible to speculate about their effects, ranging from coagulation and inflammation caused by the 26 putative platypus serine proteases, to pain caused by the stonustoxin-like (18 putative toxins) and latrotoxin-like (7 putative toxins) families [37]. This study probably uncovered the majority of platypus venom genes, but the methodology based on similarity searches using known toxins meant that it was likely to miss completely novel venom genes. In order to identify novel venom genes with no similarity to known toxins in other species, it is possible to take advantage of the seasonal nature of platypus venom production by comparing gene expression levels in active *versus* regressed glands. Genes that are highly expressed in active glands compared to regressed glands are probable toxin genes. This analysis reveals five more putative toxins unique to the platypus: growth differentiation factor 15, nucleobindin-2, complement decay-accelerating factor 55, CXC-chemokine, and corticotropin-releasing factor-binding protein [38]. In the future, functional studies are required to characterize the role of these putative toxins in platypus venom, which may reveal candidates for the discovery of novel drugs (reviewed in [39]).

### 3.5. Platypus Venom Gene Evolution

As well as identifying a wealth of new putative toxins, genomic studies of platypus venom have enabled characterization of the evolutionary origins of this trait. Ultimately, genetic origins of toxins tend to reflect the tissue origins of venom glands. Snake venom glands are specialized salivary glands, derived from the pancreas, and there are a number of snake toxins that are of pancreatic origin [40] or salivary gland origin [41]. In contrast, the platypus venom glands are thought to be derived sweat glands [17], and many of the platypus venom genes, such as the kallikreins and defensins, are derived from skin-expressed gene families [11,37].

At a proximate level, genomics has uncovered several key mechanisms acting upon platypus venom genes and venom genes in other species. Notably, gene duplication is common, with many toxin families containing multiple members [11,37]. Gene duplication occurs when a gene is replicated within the genome, for example by unequal crossing over during meiosis, to form two copies. One copy performs the original function of the gene; the second copy is then free to vary, and neofunctionalization (the development of new functions) may occur [42]. The process of gene duplication is a significant source of variation for the evolution of novel traits via either positive selection (adaptive evolution) or genetic drift. There is evidence of gene duplication in the evolution of platypus venom genes, where venom peptides have been derived via duplication from genes encoding non-toxins. For example, the OvDLPs have evolved via gene duplication from the antimicrobial beta-defensins thought to protect altricial platypus hatchlings from infection [43], after which they neofunctionalized to become toxins [11]. Gene duplication is also a source of platypus venom diversification, as it has generated large multigene families of toxins, possibly resulting in increased expression levels of a particular toxin type [36]. Gene duplication has been found to be an important process in the evolution of a number of other venoms (reviewed in [44]), particularly those of cone snails, spiders, and snakes (e.g., [45,46,47,48]).

Gene duplication alone does not explain the evolution of the platypus venom genes, revealing that additional mechanisms have played a role in venom evolution. Mechanisms that may also be important sources of new toxins include recruitment of multiple genes from the same family into venom gland expression; mutations in regulatory or coding regions; and alternative splicing [49]. These processes are important in the evolution of venoms in other species, for example in various snake venoms, which exhibit alternative splicing of an acetylcholinesterase gene [50] and modification of existing cysteine rich secretory protein and kallikrein genes [41]. These and further evolutionary processes, such as domain duplication and domain loss (reviewed in [44]), may be identified in platypus venom in the future.

Many platypus toxins have been recruited from existing non-toxin gene families, some of which have been independently co-opted as venom toxins in other species. It thus appears that there are certain protein motifs that are repeatedly selected during the evolution of venom molecules across divergent species (*i.e.*, convergent evolution) [37]. Convergent recruitment of venom toxins has been demonstrated across a wide range of venomous taxa, including hymenoptera, ticks, scorpions, spiders, cephalopods, cnidarians, cone snails, fish, shrews (reviewed in [1]), and now platypus effectively covering all major venomous phyla. These repeated, independent iterations of venom development allow us to determine the features that predispose a protein to evolve into a venom toxin: proteins with stable scaffolds that can be slightly modified to allow diversification into multimember families with differing activities; extensively cysteine cross-linked proteins; and secretory proteins (e.g., [1]). These features are displayed by many of the platypus toxins, including the OvDLPs (cysteine cross-linked) and serine protease inhibitors (a multigene toxin family).

## 4. Secondary Losses of the Venom System: The Echidnas

Based on morphological and molecular data, echidnas (*T. aculeatus* and *Zaglossus sp.*) are believed to have amphibious ancestry and have re-evolved terrestriality [8,10], although this is still debated [51]. Like the platypus, echidnas also have the anatomical elements of the crural system, thought now to represent a secondary regression of a venom system. Male *Tachyglossus* echidnas have small spurs (0.5–1.0 cm) (Figure 2), and females have vestigial spurs that are usually lost in later life [52,53]. Spurs have also been observed in *Zaglossus* males and some females, with a pus-like exudate emitted from the base of the male spur [17,52]. The spur in *Tachyglossus* and presumably *Zaglossus* is connected via a duct to the crural gland below the knee, which is active during the breeding season, and exudes a milky secretion when sliced open postmortem (reviewed in [18,52]). The echidna spur is covered by a protective skin flap from which it cannot be erected or everted. Compared to platypus spurs, echidna spurs cannot be rigidly locked into place for spurring, as they are arranged differently with respect to the attachment of the *cornu calcaris* to the *os calcaris* to the tarsal bones [14,18].

The anatomy of the echidna crural system and the seasonal activity of the crural glands, along with the lack of reports of aggressive use of spurs in the literature [18], suggest that the echidna possesses a regressed venom system (Figure 1). The crural system in modern echidnas may instead function as a scent gland [18], which is supported by chemical analyses of the waxy secretion at the base of the spurs that identified a number of large molecules with putative roles in chemical communication [54]. In addition, there is a lack of venom genes expressed in the *Tachyglossus* crural gland [55]. A transcriptomic study has revealed that the OvDLPs, the most abundant platypus venom toxins, are not expressed at all [55], although this contradicts HPLC work that did find evidence of two OvDLPs and the peptide isomerase in a sample of *Tachyglossus* crural gland exudate [56]. Only five platypus venom transcripts were found to be expressed in echidna crural gland, all at very low levels, and these may be “evolutionary remnants” [55]. It is possible that the echidna’s spiny covering reduced the need to use spurs and venom as defense, and the redundancy of the metabolically expensive venom may have then resulted in the regression of the venom system. The echidna’s transition from an aquatic to a terrestrial habitat may have allowed the use of scent glands (which are presumably less effective in watery habitats), resulting in the recruitment of the crural system for mate attraction in this usually solitary animal.

## 5. Looking Forward: Genomics and Mammalian Venom

Given the current wide availability of relatively inexpensive DNA sequencing, genomic techniques will become increasingly important in the examination of venom toxins and the elucidation of their evolutionary histories. Transcriptome sequencing (RNA-seq) has already allowed the identification of a suite of potential platypus venom toxins [37,38]. RT-PCR expression studies have enabled the characterization of platypus venom gene evolutionary pathways [35,36], and comparisons of genome sequence with transcriptome sequence data have revealed a complex history of gene duplication and recruitment into the platypus venom gland [11,49]. In the future, the availability of an echidna genome for comparison with the echidna crural gland transcriptome may allow us to identify gene losses and mutations associated with venom degeneration, providing further insight into the process of venom system regression.

Genomic techniques offer more than just the ability to fully characterize monotreme venom; they will also enable us to further understand the evolutionary history of this unusual mammalian trait. Molecular phylogenetic techniques already applied to the platypus OvDLPs [11] will enable further dating of the emergence of the platypus venom gland and echidna crural gland transcripts. It should even prove possible to reconstruct the venom genes of the ancestral monotreme in order to further characterize the evolutionary history of monotreme venom. There are bioinformatic techniques that allow prediction of the gene and amino acid sequences at ancestral nodes of phylogenetic trees (e.g., [57]), and reconstruction of ancestral venom genes in other venomous taxa has already been carried out, for example for the venom metalloproteinases of snakes [58].

These genomic techniques, in combination with proteomic and biochemical analyses, may also be applied to other poorly characterized venoms, particularly of mammals. An examination of repeated independent iterations of mammalian venom evolution will be a robust means of determining the generalities or specificities of mammalian venom development, and these comparisons can be expanded to include transitions to venom across all taxa. Under what circumstances does mammalian (or other) venom evolve? Are the same evolutionary mechanisms conserved? Do venomous mammals (and other taxa) recruit from the same ancestral non-toxin families into the venom gland, and how is this recruitment regulated? These are some of the big questions remaining in the field of venom evolution, and genomics is a powerful tool enabling us to answer them.
